# Reclamation intensifies the positive effects of warming on N_2_O emission in an alpine meadow

**DOI:** 10.3389/fpls.2023.1162160

**Published:** 2023-03-23

**Authors:** Zheng Li, Yan Li, Guozheng Hu, Hongbao Wu, Yan Liang, Jun Yan, Shicheng He, Hasbagan Ganjurjav, Qingzhu Gao

**Affiliations:** ^1^ Institute of Environment and Sustainable Development in Agriculture, Chinese Academy of Agricultural Sciences, Beijing, China; ^2^ National Agricultural Experimental Station for Agricultural Environment, Nagqu, China; ^3^ Tianjin Meteorological Bureau, Tianjin, China; ^4^ College of Resource and Environment, Anhui Science and Technology University, Fengyang, China; ^5^ Ordos Foresry and Grassland Science Institute, Ordos, China; ^6^ Nagqu Grassland Station, Nagqu, China

**Keywords:** warming, reclamation, alpine meadows, Qinghai-Tibet Plateau, Nitrous Oxide

## Abstract

Climatic warming can alter grassland nitrous oxide (N_2_O) emissions due to soil property alterations. However, how the reclamation affect grassland N_2_O flux under warming conditions remains unclear in alpine meadow ecosystems. We conducted a long-term manipulative warming experiment in a natural alpine meadow and a cultivated grassland on the Qinghai-Tibetan Plateau to explore the separate and interactive effects of warming and reclamation on the soil N_2_O emission flux. N_2_O fluxes were measured under four treatments including control (CK), warming (W), reclamation (R) and warming under reclamation (WR) from August 2018 to July 2019. We measured the content of soil C, N nutrients and 5 enzymatic activities in 2018 and 2019. Correlation analysis and structural equation modeling were used to clarify how soil N availability and soil enzyme activities affect N_2_O emission. Our results indicated that compared to the ambient conditions for the growing and non-growing seasons, soil N_2_O flux was significantly increased 59.1% and 152.0% by warming and 28.4% and 142.4% by reclamation, respectively. Compared with W, WR significantly increased N_2_O flux by 18.9% and 81.1% during the growing and non-growing seasons, respectively. Soil moisture was negatively correlated to enzymatic activity and N_2_O flux. Both warming and reclamation promoted soil nitrification by increasing related enzymatic activities that acted to increase the N_2_O flux. Reclamation resulted in a greater sensitivity of the activity of ammonia monooxygenase and hydroxylamine oxidoreductase to warming, thus enhancing the effects of warming on increasing the N_2_O flux. Our research indicated that reclamation can additionally increase the effects of warming on N_2_O emissions for alpine meadows. Therefore, excessive expansion of arable land should be avoided, and new reclamation sites should be planned scientifically, as warming is expected to intensify in the future.

## 1 Introduction

Terrestrial ecosystems account for 70% of the global N_2_O production (Barnard et al., 2005) and in particular, grasslands cover 35% of the world’s land area (Dubois, 2011) and are a primary source of this gas that is released into the atmosphere. Grasslands are facing the dual pressure of warming and reclamation and these changes can affect soil N_2_O emissions by altering soil nutrition, temperature, moisture and microbes (Contosta et al., 2011; Abraha et al., 2018).

Warming can alter soil N availability (Elrys et al., 2021; Li et al., 2022a) and associated enzymatic activities (Rich et al., 2003; Wendeborn, 2020) as well as nitrification and denitrification processes. Generally, warming can accelerate soil N cycling, and this results in increased N_2_O flux. Warming also increases turnover of soil N and soil microbe activities in ice land grasslands (Séneca et al., 2021) and this increases N loss as N_2_O (Robertson and Groffman, 2007). In the Qinghai-Tibetan Plateau (QTP), the annual N_2_O flux for the alpine meadows increases gradually with temperature due to the direct positive effects of warming on soil microorganisms and enzymes (Du et al., 2016). However, other studies have reported that warming did not increase N availability and N_2_O emissions. For example, a meta-analysis about soil carbon and nitrogen dynamics on the Tibetan Plateau reported insignificant response of soil $NH_{4}^{+}-N$ to warming in the grassland (Zhang et al., 2015). Moreover, a recent study suggested that warming decreased the soil moisture in a relatively wet soil and probably increased O_2_ concentrations thereby inhibiting N_2_O production through denitrification in an alpine meadow (Wang J. et al., 2021). In contrast, warming did not significantly affect N_2_O emission during the first and the second growing season, but stimulated N_2_O uptake in the third growing season in a tundra ecosystem on the Chinese Changbai Mountain (Zhou et al., 2016).

Reclamation is a common approach for degraded grassland restoration or forage production and this practice alters soil N and moisture content and soil N_2_O emissions (Buchen et al., 2018). Firstly, ploughing increases N input from mineralization of the organic N in plant litter resulting in increased N_2_O emission (Drewer et al., 2017). Secondly, minimum tillage increases soil N_2_O by increasing soil water-holding capacity that results a decrease in aerobic soil conditions that favors denitrification (Krol et al., 2016). Thirdly, plowing can increase soil pore spaces and air permeability and release trapped gases in soil pore spaces thus increasing soil N_2_O flux (Ball et al., 1999; Heincke and Kaupenjohann, 1999). Finally, soil organic matter mineralization can be promoted by reclamation *via* increased soil oxygen consumption thereby again favoring denitrification (Li et al., 2007). For example, a study of 3 Canadian Arctic ecosystems suggested the consumption of soil organic matter facilitated denitrification resulting in increased N_2_O production (Paré and Bedard-Haughn, 2013).

The Qinghai–Tibetan Plateau (QTP) is called the “third pole” of the world (Qiu, 2008) due to its high altitude and coldness. The alpine meadow serves as a dominant ecosystem in the QTP (Wang et al., 2014) and an important reservoir of N_2_O emissions (Hu et al., 2010). Climate warming has profoundly affected the QTP where air temperature has reached 0.25°C per decade (Yao et al., 2019). The shortage of animal forage and grassland degradation has driven the conversion of some alpine meadows into cultivated grasslands for forage (Gao et al., 2019) and the numbers of converted meadows have dramatically increased in high altitude locations over the recent decades (Yang et al., 2015). There have been numerous recent studies documenting how warming affects N_2_O emission flux in the growing season for these areas (Zhu et al., 2015; Chen et al., 2017; Zhao et al., 2017; Wang J. et al., 2021). However, the interactions of warming and reclamation on the soil N_2_O emission flux remains unclear.

We used open-top chambers (OTC) to perform a long-term manipulative experiment in a cultivated grassland and a natural alpine meadow on the QTP. We aim to explore whether there are interactive effects of warming and reclamation on grassland N_2_O emission and to clarify how soil N availability and soil enzyme activities regulate the responses of N_2_O emission to warming and reclamation. We hypothesized that (1) warming and reclamation can affect soil nutrients and increase nitrification enzyme activity to promote N_2_O flux; (2) there are significant positive interactions between warming and reclamation on soil N_2_O flux.

## 2 Materials and methods

### 2.1 Study site

Our experiment was started at 2011 in the Nagqu National Observation and Experimental Station for Agricultural Environment (31.441°N, 92.017°E; 4460 m above sea level), Tibet Autonomous Region, China. The data from the China Meteorological Data Sharing Service System indicated the mean annual temperature (-0.6°C) and the mean annual precipitation (457.6 mm) from 1980 to 2011 in this region. Here, the sedge *Kobresia pygmaea* dominates the alpine meadow as a main grassland type. *Elymus nutans* was used to build cultivated grassland in 2011, when we just plowed the soil of alpine meadow and planted the seeds of *Elymus nutans* without any fertilizer. All the experimental sites were fenced in 2010 and there were no activities of grazing, fertilization or mowing during the experimental period.

### 2.2 Experiment design

**Figure 1:**
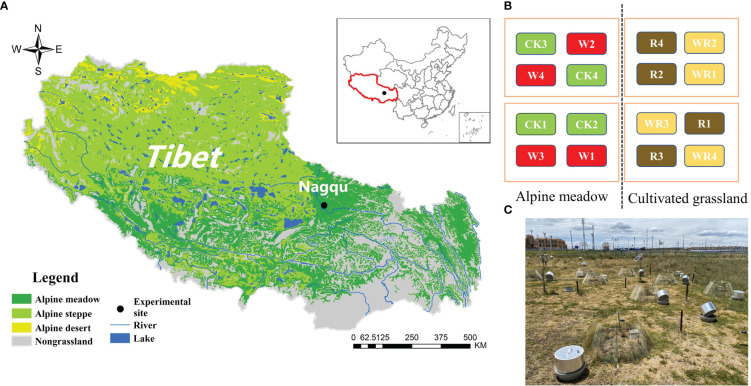
The figure of experimental site location **(A)**, plot diagram **(B)** and realistic scene**(C)**.

**Figure 2:**
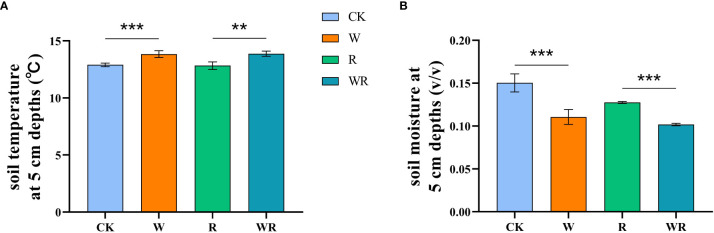
Soil temperature (°C) and moisture (v/v) at 5 cm depths under four treatments with the result of t-test. CK, control; W, warming; R, reclamation; WR, warming under reclamation. Mean ± Standard error (n = 4) was shown in the figure. (*0.01< *p*< 0.05; **0.001< *p*< 0.01; ***p< 0.001).

In this experiment, the OTCs (made of solar radiation-transmitting plastic; height 0.45 m; diameter at ground height 1.20 m and diameter at maximum height 0.65 m) were used to simulate climatic warming. The warming experiment began in 2011 with a design including four replicates and four treatments: control (CK), warming (W), reclamation (R) and warming under reclamation (WR). At each of the locations for planted grassland and meadow, there were a total of eight plots. Each test location had a surface area of 2,500 cm^2^. The plot diagram and realistic picture were showed in **Figure 1**. Measurements of soil N_2_O flux began in July 2018 and ended in August 2019. After warming, in the growing season, soil temperature (5 cm depth) significantly increased by 1.2 °C and 1.0 °C in the alpine meadow and the cultivated grassland, respectively and soil moisture (5 cm depth) significantly decreased by 5.5% and 2.6%, respectively (**Figure 2**).

### 2.3 Microclimates measurements

Throughout the growing season, the EM50 Data Collection System from Decagon Devices, Pullman, WA, USA, was utilized for soil temperature and moisture content measurements at 5 cm depths at 30 min intervals in each plot and site. During the non-growing season, no microclimatic data were gathered.

### 2.4 N_2_O flux measurements

Closed static chamber and gas chromatography were used for the measurement of N_2_O emission flux (Yan et al., 2018). To reduce the heating effect of solar radiation during sampling, heat-insulating foam was applied to the exterior of each chamber. Each plot had a cylindrical container with a shaft ring and chamber that was 31.8 cm in diameter and 20 cm high. With a headspace height of 20 cm, the collar’s bottom was placed in the ground (5 cm). Each chamber has a fan to guarantee even air distribution and a ventilation duct to maintain equilibrium throughout sampling. Gas samples (30 mL each) were taken at 0, 5, 15, and 30 minutes after the chamber was closed using pre-vacuumed 10 mL air bottles. N_2_O fluxes were sampled on all plots at 11:00 - 13:00 (during this period, the weather in this area is generally clear, and thunderstorms are avoided as much as possible to reduce the influence of weather factors on the experimental determination) once every 15 days in each month during growing season (May to September) and in the middle of each month during non-growing season (October to April). In total, we measured N_2_O flux twice in a month during growing season and once in a month during non-growing season. The N_2_O flux data for September 2018 were lost. Gas chromatography with a flame ionization detector (Model 4890D; Agilent, Wilmington, TX, USA) was used to quantify N_2_O in gas samples. The calculation of N_2_O emission flux is as shown below (Yuesi and Yinghong, 2003):


$$F_{N_{2}O}=\rho \times \frac{P}{P_{0}}\times \frac{V}{A}\times \frac{\Delta c}{\Delta t}\times \frac{273.15}{273.15+T}$$

where $F_{N_{2}O}$ is the N_2_O flux (μg m^-2^h^-1^), ρ (kg/m^3^) is the N_2_O gas density, *P* (kPa) is the atmospheric pressure at the time of sampling, *P*_0_ (kPa) is the standard atmospheric pressure, *V* (m^3^) and *A* (m^2^) are the capacity and basal area of the chamber, respectively. $\frac{\Delta c}{\Delta t}$ is the gradient of the linear regression for the gas concentration gradient with time (m^3^ m^−3^ h^−1^), and T is the mean air temperature throughout the sampling period (°C).

We used the average of two daily N_2_O emission flux as the monthly N_2_O flux for each treatment in the growing season. In the non-growing season, the daily N_2_O flux represented the monthly N_2_O flux The calculation for the mean N_2_O emission fluxes in the growing season (from June to September), non-growing season (from October to May) was the same as used for the monthly N_2_O emission fluxes. We took the mean N_2_O emission fluxes for each observation day during a particular period as the average N_2_O emission flux during that period.

### 2.5 Soil C and N measurements

A bucket auger (5 cm diameter) was used to collect soil samples of each experimental plot at 0–15 cm depth in mid-Aug 2018 and mid-July 2019. After collecting, the samples were passed through a soil sieve with 2 mm apertures. Soil total C (TC) and total N (TN) contents were determined by A Vario EL III TOC element analyzer (Elementar, Hanau, Germany). Soil organic carbon (SOC) content was determined by the potassium dichromate oxidation method. Soil samples extracted by 2 M KCl (Wei et al., 2014) were used to detect the content of $NH_{4}^{+}-N$ and $NO_{3}^{-}N$ by a flow injection auto analyzer (Auto Analyzer 3, Bran Luebbe, Norderstedt, Germany). Dissolved organic nitrogen (DON) was obtained as the difference between the TN and inorganic N ( $NH_{4}^{+}$ , $NO_{2}^{-}$ and $NO_{3}^{-}$ ) in the filtrate. Inorganic nitrogen was also measured by a flow injection auto analyzer (Auto Analyzer 3, Bran Luebbe) as previously described (Yang J. et al., 2021).

### 2.6 Soil enzymatic activity measurements

Soil ammonia monooxygenase (AMO), hydroxylamine oxidoreductase (HAO), nitrite reductase (NXR), nitric oxide reductase (NOR) and nitrous oxide reductase (NOS) were measured using enzyme-linked immunosorbent assay (ELISA) commercial kits to determine the related enzyme activities (U/g). The specific function of these enzymes involved in nitrification and denitrification are recorded in the following table (**Table 1**). In the kit, solid phase antibody was to be combined with enzyme and its labeled antibody to form antibody-antibody-enzyme-labeled antibody complex. After thorough washing and adding specific substrate, this color of complex began to change. The shade of the color was positively correlated with the activity of the sample enzyme. The absorbance (OD value) was measured with an enzyme label at 450nm wavelength, and the concentration of the activity of the sample enzyme was calculated by standard curve (Shanghai Zhuocai Biology).

**Table 1:** The main enzymes involving in soil nitrification and denitrification and their roles in specific process.

The type of enzyme	Pathway	Process
Ammonia monooxygenase (AMO)	$NH_{4}^{+}-N$ → NH_2_OH	Nitrification
Hydroxylamine oxidoreductase (HAO)	NH_2_OH → NOH	Nitrification
Nitrite reductase (NXR)	$NO_{2}^{-}$ → NO	Denitrification
Nitric oxide reductase (NOR)	NO → N_2_O	Denitrification
Nitrous oxide reductase (NOS)	N_2_O → N_2_	Denitrification

“→” means soil nitrogen changes from one form to another.

### 2.7 Statistical analyses and calculations

The effects of warming, reclamation, sampling day and their interactions on average N_2_O emission flux were tested using repeated ANOVA calculations. The average N_2_O emission flux under different treatments for the growing and non-growing seasons was calculated using the hoc-posttest with LSD (least significant difference) to examine differences among all treatments. We used two-way ANOVA to measure warming, reclamation and their interactive effects on soil nutrients and enzymatic activities in 2018 and 2019. The response ratio (r), which was computed as r=Ln(T/A) where T was the value under the treatment and A was the value under ambient conditions, was used to quantify the extent of the treatment’s impact on soil nutrients and enzyme activity. Values of the response ratio that were positive or negative showed that the therapy had either beneficial or detrimental effects. The t-test was used to determine its significant differences from zero. Linear regression analysis was used to measure the relationships between enzyme activity and N_2_O flux in the two types of grassland.

Structural equation modeling (SEM) using Amos25 SSPS software (Amos Development Corporation, IBM, Chicago, Ill, USA) was used to explore the possible causal links between N_2_O emission flux and soil environmental variables and soil enzyme activities under warming and reclamation. The indexes of the model were chosen from soil environmental variables and soil enzyme activities that were related to nitrification and denitrification. The N_2_O flux under each treatment was the average of N_2_O flux in August 2018 and July 2019. We selected the first component (65.0%) of the results of PCA for the activity of AMO, HAO, NXR, NOR and NOS to represent the enzyme activity. If the value of the root mean square (RMESA) deviation was > 0.05, the indexes and related pathways were altered until the value of RMESA was< 0.05, and the model with the lowest AIC value was selected as the best model. Before all analysis, data were checked for normality and homogeneity using F-test.

## 3 Results

### 3.1 Changes in soil N_2_O emission flux

**Figure 3:**
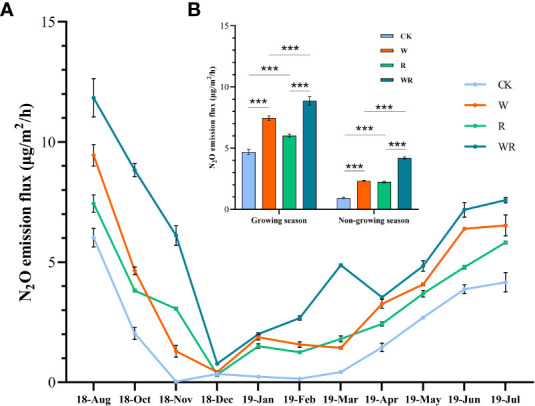
Soil N_2_O emission flux under warming and reclamation **(A)** during August 2018 and July 2019 and **(B)** different season. CK, control; W, warming; R, reclamation; WR, warming under reclamation. Mean ± Standard error (n = 4) was shown in the figure. (*0.01< *p*< 0.05; **0.001< *p*< 0.01; ***p< 0.001).

Warming, reclamation, sampling day and their interactions significantly affected the N_2_O emission flux for our experimental areas (*p*<0.01, **Table 2**). Overall, the N_2_O flux for CK was lower than that for the other treatments (**Figure 3A**). Warming significantly increased N_2_O flux by 59.1% and 152.0% (*p*<0.01) and reclamation significantly (*p*<0.01) increased the N_2_O flux by 28.4% and 142.4% during growing and non-growing seasons, respectively compared with CK. When compared with W, WR significantly (*p*<0.01) increased N_2_O flux by 18.9% and 81.0% during growing and non-growing seasons, respectively (**Figure 3B**).

**Table 2:** Results (*df*, *F* and *P*) of repeated measures analysis of variances (RMANOVA) for the separate and interactive effects of warming (W), reclamation (R), sampling day (D) on soil N_2_O emission flux.

Model	df	F	P
D	10	2244.33	<0.01
D×W	10	113.21	<0.01
D×R	10	122.97	<0.01
D×W×R	10	33.39	<0.01
W	1	1372.92	<0.01
R	1	815.41	<0.01
W×R	1	16.42	<0.01

### 3.2 Changes in soil properties

**Figure 4:**
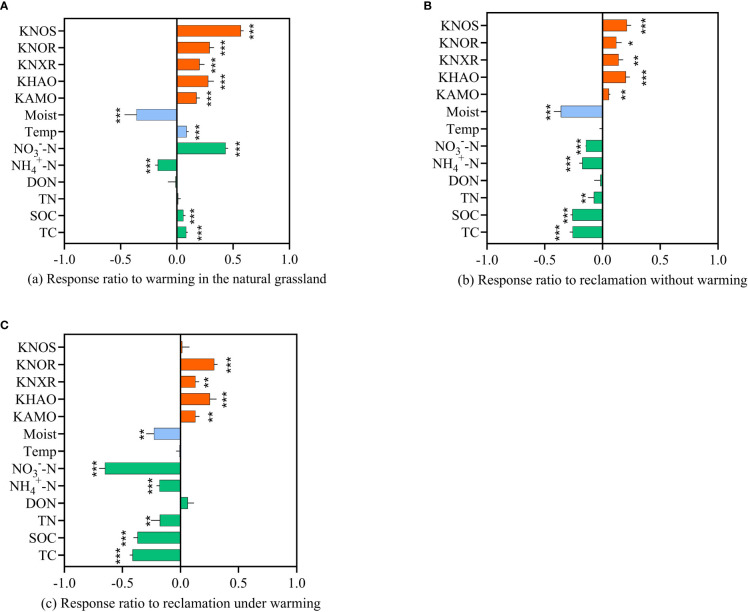
Response ratio (r) of soil properties and enzymatic activities under each treatment. **(A)** warming in natural grassland: shows the difference between CK and W, **(B)** reclamation without warming: shows the difference between CK and R, **(C)** reclamation under warming: shows the difference between W and WR. “*” indicates p < 0.05, “**” indicates p < 0.01, and “***” indicates p < 0.001. TC: soil total carbon, SOC: soil dissolved carbon concentration, TN: soil total nitrogen, DON: soil dissolved organic nitrogen, NH4+-N: soil ammonium concentration, NO3-- N: soil nitrate concentration, Temp: soil temperature, Moist: soil volumetric moisture, KAMO: enzymatic activity of AMO, KHAO: enzymatic activity of HAO, KNXR: enzymatic activity of NXR, KNOR: enzymatic activity of NOR, KNOS: enzymatic activity of NOS.

Warming significantly (*p*<0.001) increased the soil TC and SOC (**Table 3**), with the response ratio (r) values of 0.08 and 0.06, respectively in the alpine meadow (**Figure 4A**). Reclamation significantly (*p*<0.001) decreased the soil TC and SOC (**Table 3**) with r values of -0.26 and -0.26, respectively in the absence of warming (**Figure 4B**). Under warming, reclamation significantly (*p*<0.001) decreased the soil TC and SOC, with r values of -0.41 and -0.37, respectively (**Figure 4C**). Soil $NH_{4}^{+}-N$ and $NO_{3}^{-}N$ content were also significantly (*p*<0.001) affected by warming (**Table 3**), with r values of -0.17 and 0.43, respectively (**Figure 4A**). Compared with CK, reclamation significantly (*p*<0.01) decreased the soil TN, $NH_{4}^{+}-N$ and $NO_{3}^{-}N$ content (**Table 3**) with r values of -0.07, -0.17 and -0.14, respectively. Under warming, reclamation significantly (*p*<0.01 and *p*<0.001) decreased the soil TN, $NH_{4}^{+}-N$ and $NO_{3}^{-}N$ content with r values of -0.18, -0.18 and -0.65, respectively (**Figure 4C**).

### 3.3 Changes in soil enzymatic activities

Compared with CK, W significantly (*p*<0.001) increased AMO, HAO, NXR, NOR and NOS activities (**Table 3**) with r values of 0.17, 0.28, 0.20, 0.29 and 0.57, respectively (**Figure 4A**). Reclamation significantly increased AMO, HAO, NXR, NOR and NOS activities (**Table 3**) in the absence of warming with r values of 0.05 (*p*<0.01), 0.20 (*p*<0.001), 0.14 (*p*<0.01), 0.12 (*p*<0.05) and 0.21 (*p*<0.001), respectively (**Figure 4B**). Under warming, reclamation significantly increased AMO, HAO, NXR and NOR with r values of 0.13 (*p*<0.001), 0.25 (*p*<0.001), 0.13 (*p*<0.01) and 0.28 (*p*<0.001), respectively (**Figure 4C**).

**Table 3:** Results (*df*, *F* and *P*) of two-way analysis of variances (ANOVA) for the separate and interactive effects of warming (W) and reclamation effects on soil total carbon (TC), soil dissolved carbon concentration (SOC), soil total nitrogen (TN), soil dissolved organic nitrogen (DON), soil ammonium concentration ( $NH_{4}^{+}-N$ ), soil nitrate concentration ( $NO_{3}^{-}N$ ), and the enzymatic activity of AMO, HAO, NXR, NOR and NOS (KAMO, KHAO, KNXR, KNOR, KNOS).

Factors		W				R				W×R			
Year		2018		2019		2018		2019		2018		2019	
variables	df	F	P	F	P	F	P	F	p	F	P	F	P
TC	1	78.56	<0.001	371.92	<0.001	1350.5	<0.001	2011.1	<0.001	1.95	0.188	549.52	<0.001
SOC	1	61.07	<0.001	135.91	<0.001	1026.8	<0.001	930.83	<0.001	6.21	0.028	214.27	<0.001
TN	1	114.25	<0.001	28.17	<0.001	32.11	<0.001	18.41	0.001	7.19	0.020	28.17	<0.001
DON	1	0.08	0.786	11.04	0.006	0.33	0.575	0.42	0.528	1.22	0.292	1.09	0.317
$NH_{4}^{+}-N$	1	70.47	<0.001	258.54	<0.001	99.26	<0.001	216.76	<0.001	3.21	0.098	20.25	0.001
$NO_{3}^{-}N$	1	425.81	<0.001	92.02	<0.001	841.17	<0.001	617.48	<0.001	479.7	<0.001	245.53	<0.001
KAMO	1	426.65	<0.001	115.41	<0.001	87.22	<0.001	22.66	<0.001	46.621	<0.001	0.03	0.857
KHAO	1	107.09	<0.001	347.83	<0.001	41.77	<0.001	243.12	<0.001	26.579	<0.001	0.01	0.909
KNXR	1	104.61	<0.001	35.55	<0.001	105.46	<0.001	1.66	0.222	0.077	0.786	0.13	0.729
KNOR	1	298.19	<0.001	202.79	<0.001	108.96	<0.001	61.49	<0.001	21.039	0.001	30.99	<0.001
KNOS	1	144.37	<0.001	462.15	<0.001	12.56	0.004	8.26	0.014	23.432	<0.001	0.05	0.825

### 3.4 Factors affecting soil N_2_O emissions

**Figure 5:**
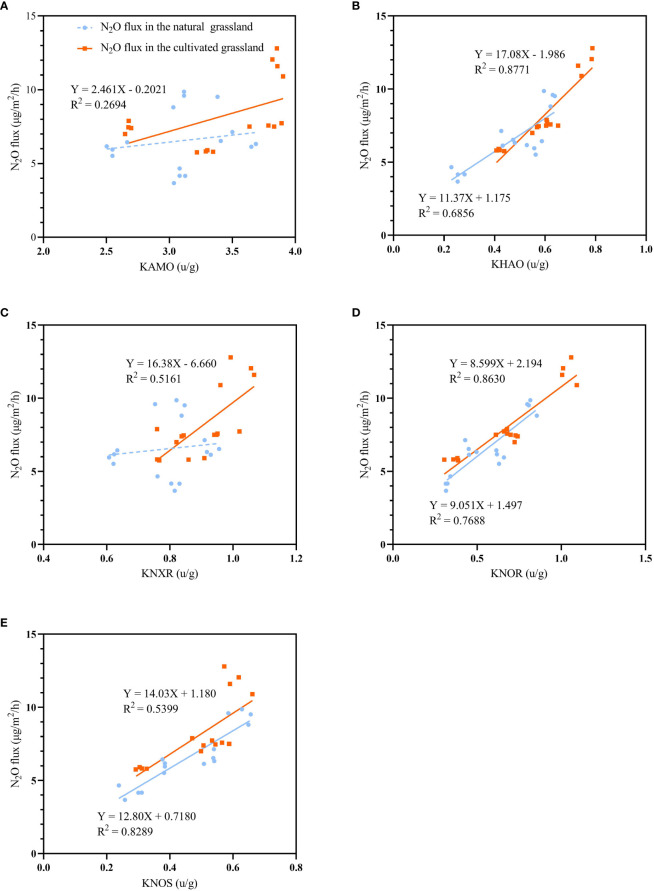
Linear regression between N_2_O emission flux and enzymatic activity of AMO **(A)**, HAO **(B)**, NXR **(C)**, NOR **(D)** and NOS **(E)** (KAMO, KHAO, KNXR, KNOR, KNOS) in natural alpine meadow (blue dot and line) and cultivated grassland (orange square and line). Solid line indicates *p*< 0.05 and dotted line indicates *p* > 0.05.

In the natural alpine meadow, AMO and NXR activities were not significantly correlated with N_2_O flux. In the cultivated grassland, AMO, HAO, NXR, NOR and NOS activities were all significantly (*p*<0.05) correlated with N_2_O flux. Moreover, after reclamation, the N_2_O flux was more sensitive to the activity of HAO (**Figure 5**).

**Figure 6:**
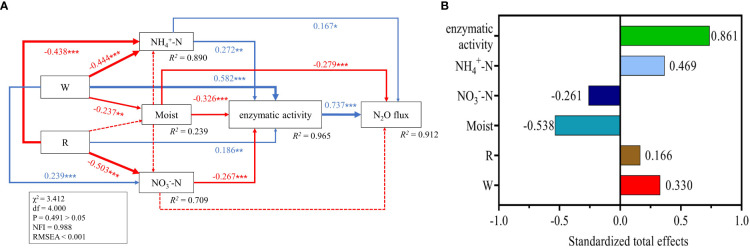
Direct and indirect effects of warming (W) and reclamation (R) on N_2_O emission flux **(A)**. W, warming; R, reclamation, $NH_{4}^{+}-N$ : soil ammonium concentration, $NO_{3}^{-}N$ : soil nitrate concentration, Moist: soil volumetric moisture, enzymatic activity: the first principal component of PCA analysis for the activities of AMO, HAO, NXR, NOR and NOS. The arrows’ numbers correspond to path coefficients. Beneficial and detrimental correlations are denoted by blue and red arrows, respectively. At the bottom right of boxes, the related *R* square in the model were displayed. “*” tells *p*< 0.05, “**” tells *p*< 0.01, and “***” tells *p<* 0.001. Standardized total effects **(B)** were showed to evaluate the effects of W, R, Moist, $NH_{4}^{+}-N$ , $NO_{3}^{-}N$ and enzymatic activity on N_2_O flux in the model, and the plus or minus of these values represented their positive or negative effects respectively on N_2_O flux. The information presented here includes all data gathered throughout treatments and years (n = 32).

Structural equation modeling indicated that warming and reclamation did not directly affect the N_2_O emission flux. Warming increased soil enzymatic activity (0.58) and decreased soil $NH_{4}^{+}-N$ content (-0.44) and soil moisture (-0.24). Reclamation increased the soil enzymatic activity (0.19) and decreased soil $NH_{4}^{+}-N$ (-0.44). Soil enzymatic activity positively (0.74) affected the soil N_2_O flux but was negatively (-0.28) affected by soil moisture. Soil $NH_{4}^{+}-N$ promoted enzymatic activity (0.27) while soil moisture (-0.33) was negatively correlated to soil enzymatic activity (**Figure 6A**). The standardized total effect of warming and reclamation on the N_2_O flux were 0.33 and 0.17, respectively (**Figure 6B**). Overall, the indirect effects of warming and reclamation *via* changing soil $NH_{4}^{+}-N$ content, soil $NO_{3}^{-}N$ content, soil moisture and soil enzymatic activity explained 91.2% of the variance in N_2_O emission flux.

## 4 Discussion

### 4.1 Effects of warming and reclamation on soil nutrients

Increasing temperatures can increase soil carbon and nitrogen cycling in cold ecosystems and warming has increased soil C and N content in the QTP terrestrial ecosystems (Chang et al., 2021; Huai Chen et al., 2013). In our study, warming significantly increased soil TC and SOC in the alpine meadow, which might be explained by the increase of living fine root biomass and root exudation rates under warming, thus promoting C input from root exudates C into the soil (Liu et al., 2022; Wang Q. et al., 2021). Warming can facilitate nitrification (Bai et al., 2013) and directly increase related enzymatic activities by increasing soil temperature (Du et al., 2016). We observed that warming significantly decreased soil $NH_{4}^{+}-N$ while increasing soil $NO_{3}^{-}N$ as well as AMO and HAO activities in the alpine meadow (**Figure 4A**). However, our results were not consistent with the general effects of warming on soil SOC and $NH_{4}^{+}-N$ . For an instance, previous studies thought the rough balance of the increase of both plant input and soil microbial respiration by warming led to the insignificant response of C, N contents to the impacts of warming (Zhang et al., 2015). This difference might be explained by discrepant plant and soil microbial composition, even in similar ecosystem. The actual mechanism by which warming affected soil C, N in the alpine meadow should be further investigated.

Generally, conversion of alpine pastureland to artificial grassland decreases soil C and N (Linsler et al., 2013). Firstly, reclamation can destroy the soil macroaggregate (Liu et al., 2014) that is critical to soil nutrient distribution and sequestration rates (Kasper et al., 2009) thus causing the loss of soil C and N (Krol et al., 2016; Zhang et al., 2021). Secondly, cultivation may lead to a loss of soil organic matter in the surface soil layers due to the increase of soil C and N mineralization under tillage (Busari et al., 2016). Our results confirmed that compared with CK, there was a significant decline of soil TC, SOC, TN, $NH_{4}^{+}-N$ and $NO_{3}^{-}N$ after reclamation.

### 4.2 Effects of warming and reclamation on N_2_O flux

Soil N content serves as an important factor affecting soil N_2_O emissions (Li et al., 2022b). We found that the content of soil $NH_{4}^{+}-N$ and not $NO_{3}^{-}N$ , was positively correlated with N_2_O flux. Previous studies of relationships between soil N_2_O emission and soil moisture have mixed results. A global meta-analysis indicated a strong positive relationship between the effect sizes of N_2_O emission and soil moisture (Li et al., 2020) although another study found no significant correlation between soil moisture and N_2_O emission in the alpine steppes of the QTP (Yang Y. et al., 2021). However, our results demonstrated that soil moisture was negatively correlated to enzymatic activities and N_2_O flux. This was most likely the result of increased activity and abundance of nitrobacteria that resulted from the modest decrease of soil moisture (Liu et al., 2017).

Reclamation also loosens the soil and enhances its total porosity to increase soil oxygen content (Farahani et al., 2022) that could promote nitrification (Wendeborn, 2020) leading to increased soil N_2_O production. Significant increases of alpine meadow soil N_2_O emissions have been documented following cultivation (Zhang et al., 2017). This was consistent with our study results that indicated reduced soil N_2_O fluxes for the control treatment than that under reclamation (**Figure 3**). Accordingly, we observed the positive effect of reclamation on enzymatic activity in the model (**Figure 6**).

Agricultural land use can alter the resilience of the ecosystem towards warming such as elevated N_2_O emissions demonstrated for the Amazon (Lage Filho et al., 2022). We found a significant interaction of warming and reclamation on N_2_O flux primarily due to their interactions with soil moisture. Soil moisture in cultivated grasslands is usually less than that in the natural grassland (Ganjurjav et al., 2018) and it can be reduced further by warming. We observed WR significantly decreased soil moisture compared to W (**Figure 4C**). Therefore, the decrease of soil moisture by the synergistic effect of warming and reclamation might create suitable environmental conditions for nitrification to increase N_2_O emissions that also might serve as a primary contributor for the AMO, and HAO activity increases we found in our study. We confirmed that soil $NH_{4}^{+}-N$ , moisture and enzymatic activity were altered by warming and that reclamation promoted nitrification and increased soil N_2_O flux.

Soil N_2_O is predominantly produced by microbial-mediated nitrification and denitrification (Doltra et al., 2015). In alpine meadows, soil N_2_O is primarily produced through nitrification rather than denitrification in the growing season (Du et al., 2016; Yan et al., 2018). In our study, the higher N_2_O emission flux under warming and reclamation was primarily derived from the increase of enzymatic activity related to nitrification. During the non-growing season, denitrification is a primary N_2_O production process because of snow cover and freezing-thawing can create an anaerobic environment in topsoil (Priemé and Christensen, 2001; Groffman et al., 2006; Risk et al., 2013). For instance, warming massively increased N_2_O emissions (101.9%) during the non-growing season in an alpine grassland of the Tianshan (Gong et al., 2021). Analogously, we found that warming significantly increased the average N_2_O emission flux during non-growing season in both ambient and reclamation conditions (**Figure 3**). This may be due to the increased denitrification enzymatic activities and soil nitrogen mineralization rates (Contosta et al., 2011). However, we did not measure soil properties and related enzymatic activities during the off-growing season. The mechanism for soil N_2_O flux response to warming and reclamation during the non-growing season still needs further investigation.

### 4.3 Uncertainties and implications

The primary uncertainty for our experimental design was in the warming chamber since temperature as well as evapotranspiration and gas exchange can be altered in open-top chambers. The rain sheltering effect resulted in lower levels of precipitation reaching the warming chambers than for the ambient temperature chambers and could directly affect soil N_2_O flux in the chambers. Nevertheless, the above-mentioned methods are acceptable because warming chambers have been widely used in grassland ecosystems to simulate warming (Chen et al., 2016; Zhao et al., 2017). Moreover, we have a tiny database and are unable to undertake automated continuous measurement of soil N_2_O flow in warming chambers. It makes it difficult to draw firm conclusions for complete alpine meadow ecosystems on the QTP.

Our results can provide insights for future research and grassland management as follows: (1) despite the increase of enzymatic activities by reclamation and warming, the question of whether more soil N will be lost as N_2_O or be incorporated by plants requires the isotope tracer technique for an answer; (2) it is necessary to study the mechanism by which reclamation and warming increase the soil N_2_O flux during non-growing season; (3) in response to a warming climate, no-tillage seeding may be a better way to improve the productivity of the grassland in alpine meadows due to increased N_2_O flux by reclamation in cultivated grasslands.

## 5 Conclusions

We conducted a long-term manipulative warming experiment in an alpine meadow and a farmed grassland on the QTP and found that both warming and reclamation increased the N_2_O emission flux during both the vegetation seasons and off-growing seasons. Reclamation intensified the positive effects of warming on N_2_O flux, due to their interaction on the decrease of soil moisture to increase ammonia monooxygenase and hydroxylamine oxidoreductase activities. However, warming, reclamation and their interactions on N_2_O flux during non-growing season still needs further investigation. Our results suggest that future research should pay more attention to N_2_O emissions from cultivated grasslands and the cultivated grassland in alpine meadow should be scientifically projected to better adapt to a warming climate.

## Data availability statement

The original contributions presented in the study are included in the article/supplementary material. Further inquiries can be directed to the corresponding author.

## Author contributions

The research was designed by HG and GQ. The experiments were performed by ZL, YLi, HW, YLia, JY, SH. Date analysis and draft writing were conducted by ZL and HG. The manuscript was revised by ZL and HG. All authors contributed to the article and approved the submitted version.
